# Quality Improvement Project on the Use of Clinical Global Impression (CGI) Scores in a Female Adult Inpatient Mental Health Ward

**DOI:** 10.1192/bjo.2024.407

**Published:** 2024-08-01

**Authors:** Uchechukwu Okwu, Tharindi Senasinghe, Adeola Akinola

**Affiliations:** Pennine Care NHS Foundation Trust, Manchester, United Kingdom

## Abstract

**Aims:**

Clinical Global Impression Scale (CGI) is a recognised scoring system used to assess patients across a variety of medical specialties. In this study we aim to evaluate the use of the severity scale (CGI-S) in a female adult mental health unit. We aim to explore how frequently it is used to assess patients on admission and discharge and investigate its utility in predicting a variety of patient outcomes. We hypothesise that gaining a greater understanding of the significance of CGI-S scoring can enhance in-patient care by offering insights into factors such as probable length of stay and potential benefits of in-patient admission.

**Methods:**

Patient data was collected retrospectively for the last 60 patients discharged. The resulting population data from an inpatient female ward which was then analysed using Microsoft Excel and Jamovi.

**Results:**

59 patients were included in the final data set. Population age at time of admission ranged from 18–68 years with a mean age of 38. The mean length of stay was found to be 40.2 days. 80% of patients had an admission CGI-S recorded with a mean score of 2.77. 71% had a discharge CGI-S recorded with a mean score of 1.79. 58% of patients had both admission and discharge CGI-S score recorded. The key findings of the study were a mean reduction of 1.09 in CGI-S scores, indicating an overall improvement in patient presentation by the point of discharge. Length of admission was increased by 14 days per 1 integer increase in admission CGI-S score. The data also suggests that the correlation between admission CGI-S and length of stay is statistically significant (p value of 0.016). It was also noted that patients with a discharge diagnosis of ‘Emotionally Unstable Personality Disorder’ had a smaller reduction in CGI-S score at point of discharge and required shorter hospital stays, compared with other diagnoses.

**Conclusion:**

The results of this study imply that use of CGI-S scoring in adult inpatient units is beneficial. However, its value can be better seen with improved adherence to regular completion of scores during patient reviews and is an important step to prioritise. Increase in utilisation of this tool will also likely provide clinicians with guidance in predicting which patients are likely to benefit from lengthier admissions and those that might fare better with community support.

